# Overpotential from Cosolvent Imbalance in Battery Electrolytes: LiPF_6_ in EMC:EC

**DOI:** 10.1021/acsomega.3c02088

**Published:** 2023-05-26

**Authors:** Taeho Jung, Andrew A. Wang, Charles W. Monroe

**Affiliations:** † Department of Engineering Science, University of Oxford, Parks Road, Oxford, OX1 3PJ, U.K.; ‡ The Faraday Institution, Becquerel Avenue, Harwell Campus, Didcot, OX11 0RA, U.K.; ¶ Department of Chemical Engineering, Columbia University, 500 West 120th Street, New York, New York 10027, United States

## Abstract

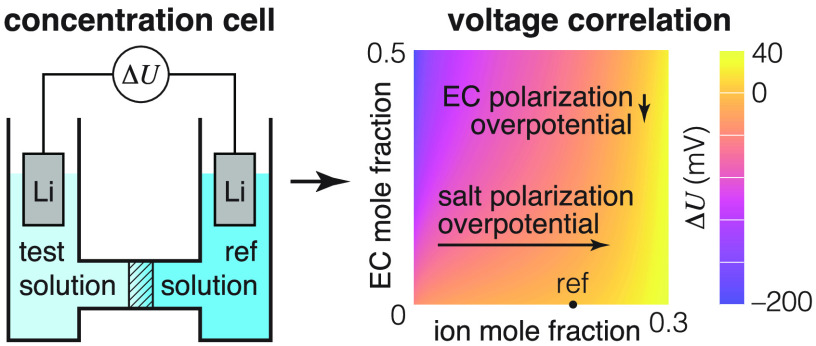

Most liquid lithium-ion-battery electrolytes incorporate cosolvent blends, but the dominant electrochemical transport models adopt a single-solvent approximation, which assumes in part that nonuniform cosolvent ratios do not affect cell voltage. For the popular electrolyte formulation based on ethyl-methyl carbonate (EMC), ethylene carbonate (EC), and LiPF_6_, we perform measurements with fixed-reference concentration cells, finding appreciable liquid-junction potentials when only the cosolvent ratio is polarized. A previously reported junction-potential correlation for EMC:LiPF_6_ is extended to cover much of the ternary composition space. We propose a transport model for EMC:EC:LiPF_6_ solutions grounded in irreversible thermodynamics. Thermodynamic factors and transference numbers are entwined in liquid-junction potentials, but concentration-cell measurements determine observable material properties we call junction coefficients, which appear in the extended form of Ohm’s law that accounts for how composition changes induce voltage drops. Junction coefficients of EC and LiPF_6_ are reported and illustrate the extent to which ionic current induces solvent migration.

## 1 Introduction

Almost every battery technology hinges on mass transport within a liquid, gel, or solid electrolyte.^1^ Today’s lithium-ion batteries rely on liquid electrolytes with moderate to high salt concentrations that include multiple solvents.^2,3^ Lithium-ion electrolytes are usually formulated at the conductivity maximum, near 1 M for nonaqueous solutions of LiPF_6_.^4−6^ Cosolvent blends based on multiple miscible uncharged components help to tailor solution properties: the maximum ionic conductivity is raised by adding a salt-dissociating polar cyclic carbonate, such as ethylene carbonate (EC); viscosity is lowered by adding one or more linear carbonates, such as ethyl-methyl carbonate (EMC). Many popular contemporary electrolyte formulations are based on cosolvent EMC:EC blends, including 7:3 EMC:EC by weight^7^ or 7:3 EMC:EC by neat-solvent volume at ambient temperature.^8^ In such blends both constituents of the EMC:EC cosolvent have comparable molarities, preventing the identification of a single molecular species within the electrolyte as a “solvent” in great excess.

Accurate transport modeling is impeded by the highly concentrated and multicomponent constitution of lithium-ion battery electrolytes. The Nernst–Planck dilute-solution theory of electrolytic transport neglects both ion–ion interactions and thermodynamic nonideality.^9^ By contrast, Newman’s concentrated-solution theory,^10^ based on the Onsager–Stefan–Maxwell equations from irreversible thermodynamics,^11^ accounts for diffusional drag interactions between ions and the nonideal concentration dependences of diffusion driving forces at higher salt molarities. These features ensure the thermodynamic consistency of Newman’s approach, as well as giving it better predictive power for engineering purposes.^12^ Physics-based modeling of lithium-ion batteries is fairly advanced, with several computational packages and databases available to facilitate the deployment of pseudo-two-dimensional (P2D) models based on porous electrode theory, like the one pioneered by Doyle et al.^13−16^ Cell-level P2D models typically describe electrolyte transport within the framework of concentrated-solution theory.

Despite the advanced state of P2D battery modeling, almost every implementation for lithium-ion batteries has neglected the multicomponent nature of the electrolyte’s solvent.^17−19^ Parametrization efforts focused on transport within the electrolyte also typically treat solvent blends as a single entity.^5,20−24^ This ‘single-solvent approximation’ is ubiquitous, despite the fact that solvent mobility has increasingly been recognized as a factor controlling battery performance.^25,26^ Several groups have observed that applied currents can drive bulk motion of both polymer and liquid electrolytes, showing that electrically uncharged solvent molecules can be driven to migrate.^27−30^

For the EMC:EC:LiPF_6_ ternary system, which we investigate here, Wang et al. used Hittorf experiments to examine current-induced polarization of the cosolvent concentration ratio.^31^ They observed significant solvent migration under applied currents, with the EMC and EC components moving at much different rates. They also observed LiPF_6_ and EMC concentration polarization that opposed the EC polarization, showing that understanding of local solvation structures derived from measurements of systems at equilibrium may not entirely carry over to dynamical situations. Wang’s experiments specifically refute the mass-transfer aspects of the single-solvent approximation for the EMC:EC:LiPF_6_ system.

There remains a need to address the electrical aspect of the single-solvent approximation—the assumption that individual solvent transport within cosolvent electrolytes does not affect their current–voltage relations. In particular, there is sparse literature evaluating whether and how local cosolvent composition affects concentration overpotentials in lithium-ion electrolytes. This paper aims to close the gap by providing a set of overpotential measurements across the EMC:EC:LiPF_6_ ternary composition space, as well as outlining a transport model based on concentrated-solution theory able to incorporate such data.

The electrolyte properties that determine how composition gradients lead to concentration overpotential include thermodynamic factors and transport coefficients.^10^ One standard method to quantify thermodynamic factors is based on measuring the so-called liquid-junction potential—the voltage drop across a concentration cell that brings two solutions, with identical constituents but different component concentrations, into contact.^6,21−23,32−34^ In the standard concentration-cell experiment, two reservoirs containing solutions with different component concentrations are connected via a porous frit, which allows electrochemical contact while impeding interdiffusion. The concentration difference induces species electrochemical-potential differences across the frit, which can be quantified by measuring the voltage drop between similar reference electrodes immersed in the two reservoirs. If the energy dissipation due to interdiffusion is negligible, the potential so measured is a state property, dependent only on the electrolyte compositions at either end of the liquid junction. The first section of this paper uses concentrated solution theory to establish how liquid-junction-potential correlations convey information about thermodynamic factors and transference for the EMC:EC:LiPF_6_ system.

Protocols for concentration-cell experiments have been described by Nyman et al.^22^ and Hou and Monroe,^34^ among others. More precise measurements can be obtained using the ‘shifting-reference concentration-cell’ method recently reported by Wang et al.^6^ In the latter sections of the paper we report fixed-reference liquid-junction potential data measured with lithium metal electrodes for EMC:EC:LiPF_6_ solutions to probe the single-solvent approximation, develop a correlation that can be employed to calculate liquid-junction potentials for a given difference in ternary composition, and apply the correlation to investigate solvent transference for this practically important battery electrolyte.

## 2 Flux Laws for Cosolvent Electrolytes

Van-Brunt et al. recently put forward a framework for extending concentrated-solution theory to cover locally electroneutral multicomponent electrolytic solutions at constant absolute temperature *T*.^35^ In a worked example, they analyzed an electrolyte comprising two solvents and a single simple salt. That calculation was used as a starting point to create a transport model for the cosolvent lithium-ion-battery electrolyte EMC:EC:LiPF_6_. Let subscripts 0, o, +, and – indicate quantities that describe the EMC, EC, Li^+^, and PF_6_^–^ species, respectively, and subscript e denote the neutral LiPF_6_ component formed by association of the Li^+^ and PF_6_^–^ ions. Transport laws for the four total species fluxes , with *i*∈{0,o,+,−}, can be expressed in terms of three component concentrations *c*_*j*_, with *j*∈{0,o,e}, the ionic current density *i⃗*, the EMC component velocity , and the respective chemical potentials μ_o_ and μ_e_ of the EMC and LiPF_6_ components, as
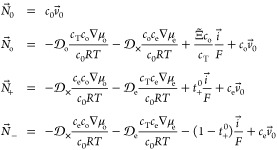
1in which *c*_T_ = *c*_0_ + *c*_o_ + 2*c*_e_ is the total species concentration, *F* is Faraday’s constant, and *R* is the gas constant. These flux laws include five transport properties: component diffusivities in EMC for EC and LiPF_6_,  and , respectively; the EC/LiPF_6_ cross-diffusivity in EMC, ; the cation transference number relative to the EMC velocity, *t*_+_^0^; and the electro-osmotic coefficient of EC in EMC, Ξ̃.^50^ To accommodate the electrical response of the EMC:EC:LiPF_6_ ternary system within the model, eqs 1 are accompanied by a flux law for charge, more specifically, an additional constitutive law showing how the potential Φ measured by a reference electrode reversible only to Li^+^ redox varies with current and composition. According to Van-Brunt et al.,^35^ a thermodynamically consistent MacInnes equation—a modified form of Ohm’s law that accounts for concentration overpotential—can be written for this system as
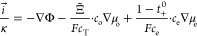
2in which the sixth and final transport property κ represents the ionic conductivity. The paper by Van-Brunt et al. also provides formulas that map the six transport properties introduced here invertibly into the Stefan–Maxwell diffusivities that parametrize pairwise drag interactions between species. Thus, the physical content of the flux-explicit model embodied by eqs 1 and 2 is identical to that of the force-explicit Onsager–Stefan–Maxwell equations on which Newman founded concentrated-solution theory.^10,35^

Because they derive from the Onsager–Stefan–Maxwell theory, the transport laws in eqs 1 and 2 rely on a set of thermodynamic state equations, which ensure that the model consistently incorporates the composition dependences of equilibrium solution properties.^36^ Two independent state equations quantify how gradients of the component particle fractions *y*_o_ = *c*_o_/*c*_T_ and *y*_e_ = *c*_e_/*c*_T_ translate into chemical-potential gradients of EC and LiPF_6_,
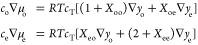
3in which four dimensionless thermodynamic factors *X*_oo_, *X*_oe_, *X*_eo_, and *X*_ee_ have been introduced to quantify nonideal variation of the component activities with respect to composition.^51^ When a cosolvent electrolyte’s mixing is thermodynamically ideal, *X*_*ij*_ = 0 for all *i* and *j*. Van-Brunt et al. provide a thermodynamically consistent parametrization of the nonideal corrections,^35^ which shows that the four *X*_*ij*_ introduced here must be constrained such that
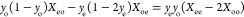
4a Maxwell relation that also follows from the “core potential” framework of Goyal and Monroe^37^ after assuming local electroneutrality. Thus, three independent state functions quantify the composition dependences of the chemical potentials μ_o_ and μ_e_. For example, when performing thermodynamic characterization of *X*_*ij*_(*y*_o_,*y*_e_) via vapor–liquid equilibrium, boiling-point elevation, freezing-point lowering, or other colligative-property measurements, one can take *X*_oo_, *X*_ee_, and *X*_eo_ to be primary quantities, and then derive *X*_oe_ from the Maxwell relation. Equation 4 reveals that cross terms must vanish, i.e., *X*_eo_ = *X*_oe_ = 0, in situations where *y*_o_ = 0 or *y*_e_ = 0, ensuring compatibility of the ternary thermodynamic model with typical binary-solution models along those edges of the composition space. The equation also suggests that *X*_eo_ = *O*(*y*_e_) or higher and *X*_oe_ = *O*(*y*_o_) or higher, since thermodynamic consistency demands that both *X*_ee_ and *X*_oo_ approach finite constants at infinite dilution.

Equations 3 define thermodynamic factors over a particle-fraction composition basis. This is convenient for characterization experiments because particle fractions are independent of temperature and pressure, and can be determined very precisely with gravimetric measurements. Flux laws are more commonly formulated in terms of molar concentrations, however. Converting fractional amounts to densities requires information about solution volume, necessitating a third thermodynamic state equation to close the model. For isothermal, isobaric, locally electroneutral EMC:EC:LiPF_6_ solutions, one can write a volume-explicit equation of state in two ways, as
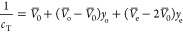
5or
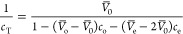
6in which  is the partial molar volume of component *i*. Component partial molar volumes generally vary with temperature and external pressure, as well as composition, dependences relatively straightforward to establish with densitometry,^10,34^ as described in Appendix A. Note that just two composition variables suffice to determine the total molarity of a ternary phase; one can either use eq 5 to write a state function *c*_T_ = *c*_T_(*y*_o_,*y*_e_) or eq 6 to write *c*_T_ = *c*_T_(*c*_o_,*c*_e_). Further rearrangement of the state functions and incorporation of the Gibbs–Duhem equation for volume^37^ show that the transformation
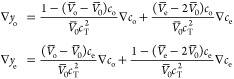
7in which *c*_T_(*c*_o_,*c*_e_) by eq 6, converts molarity gradients into particle-fraction gradients. Observe that one can obtain an inverse mapping that sends particle-fraction gradients into molarity gradients by inverting eq 7 to resolve the concentration gradients, then replacing *c*_*i*_ with *c*_T_*y*_*i*_ in the coefficient matrix and taking *c*_T_(*y*_o_,*y*_e_) from eq 5.

The molar flux laws in eq 1, MacInnes eq 2, chemical-potential constitutive laws 3, and composition constitutive laws 6 and 7 provide a thermodynamically and kinematically consistent description of locally electroneutral mass transport and charge flow within the EMC:EC:LiPF_6_ ternary system. The model is parametrized by the three partial molar volumes , ,  in the state equations accounting for solution density, three independent thermodynamic factors *X*_oo_, *X*_ee_, *X*_eo_ that describe excess mixing energetics in the chemical potential constitutive laws, and in the transport equations, three thermodynamic diffusivities , , and , the electro-osmotic coefficient of EC relative to EMC, Ξ̃, the cation transference number relative to EMC, *t*_+_^0^, and the ionic conductivity κ. At constant temperature and pressure, each of these 12 properties depends at most on the molar concentrations of EC and LiPF_6_, *c*_o_ and *c*_e_, respectively.

Although the model outlined above is thermodynamically rigorous and ostensibly complete, the presence of 12 distinct composition-dependent parameters that are convoluted within the flux laws poses a major practical challenge. The model can be tied more closely to macroscopic observations by putting some of the constitutive relations together and redefining parameters. For example, combining eqs 1, 3, and 7 produces a set of laws more akin to the Nernst–Planck equations or Newman’s transport laws for binary electrolytes:
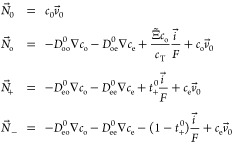
8These transport laws introduce four Fickian diffusivities relative to convection at the EMC velocity, *D*_*ij*_^0^, to stand alongside the properties Ξ̃ and *t*_+_^0^ defined earlier. Appendix B shows how previously established properties contribute to these Fickian diffusivities.

Equations 8 have a structure very similar to the laws recently used by Wang et al. to model excess molar fluxes during Hittorf experiments on the EMC:EC:LiPF_6_ system.^31^ They used Hittorf measurements to quantify Ξ̃ and *t*_+_^0^, and also provided estimates for *D*_*ij*_^0^ under the assumption that *D*_eo_^0^*c*_o_ = *D*_oe_^0^*c*_e_. Equations from Appendix B show that if mixing is thermodynamically ideal and the EC and LiPF_6_ (o and e) components are both very dilute, then *D*_eo_^0^*c*_o_ = 2*D*_oe_^0^*c*_e_; outside of this regime there is no obvious relationship that expresses how Fickian cross-diffusivities reflect the thermodynamic Maxwell relation and the symmetry of elementary transport coefficients demanded by Onsager reciprocity.^36^ Despite this issue, the symmetry of Fickian diffusivities assumed by Wang et al. does not impact their reported measurements of Ξ̃ and *t*_+_^0^.

This paper provides a set of experimental results for liquid-junction potentials that can be used to parametrize the MacInnes equation—the modified form of Ohm’s law. Putting eqs 2 and 3 together yields a more concrete statement of the MacInnes equation,
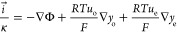
9in which κ is the previously defined ionic conductivity, but new parameters *u*_o_ and *u*_e_ appear, which we respectively call the “junction coefficients” of EC and LiPF_6_. At a given composition, the junction coefficient *u*_*i*_ quantifies how liquid-junction potential changes with respect to the particle fraction of component *i*, leaving all the other component fractions save *y*_0_ constant. Junction coefficients are dimensionless and relate to previously introduced properties through
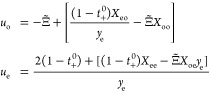
10Observe that neither of these relationships involves the diffusivities , , or —generally, junction coefficients only contain information about mixing thermodynamics and migration.

When applied to concentrated, thermodynamically nonideal electrolytic solutions, open-circuit measurements of liquid-junction potentials directly probe junction coefficients, rather than thermodynamic factors or migration coefficients. Suppose that a concentration cell is set up with one reservoir at a reference EMC:EC:LiPF_6_ composition **y**^ref^ = [*y*_o_^ref^,*y*_e_^ref^], and the other reservoir at a test composition **y**^test^ = [*y*_o_, *y*_e_], with lithium-metal electrodes (reversible only to Li^+^) immersed in each reservoir. Under open-circuit conditions ( uniformly), the potential difference between these reference electrodes at open circuit can be calculated by integrating eq 9 along a path that travels from the solution at the surface of one electrode to the solution at the surface of the other. Thus, the liquid-junction potential Δ*U* relative to the reference composition is given by
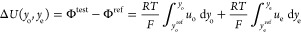
11a path integral across the composition space from point **y**^ref^ to point **y**^test^. Despite the fact that thermodynamic factors cannot be directly extracted from concentration-cell experiments for a binary salt in two solvents, cf. eq 10, measurements of Δ*U* can be made readily, using the same experimental procedure typical for one-solvent systems.^34^ It should be noted that when there is an additional solvent, the liquid-junction potential function Δ*U* lies above a two-dimensional composition space, so parametrization is not as straightforward as it would be for a one-solvent system, as conveyed by Mistry and Srinivasan.^38^ Of primary concern here, the liquid-junction potential associated with differences in solvent ratio is hard to predict *a priori*.

Although the model in eqs 7, 8, and 9 is not expressed in terms of the most fundamental properties (Onsager diffusivities, thermodynamic factors, etc.) it still contains the correct number (12) of independent properties necessitated by thermodynamic and irreversible-thermodynamic considerations. From a philosophical perspective, it is worth pointing out that the practical exercise of calculating the open-circuit voltage (OCV) across an electrolyte in a given state—the most important task for battery modeling—does not require one to isolate the distinct contributions made by thermodynamic factors, excluded-volume effects, and elementary transport properties. The structure of the simplified model equations nevertheless ensures that the observable properties (Fickian diffusivities, junction coefficients, etc.) relate to the elementary properties from Onsager–Stefan–Maxwell theory through a set of known invertible maps. Thus, if a complete parametrization of eqs 7–9 is carried out, that information can be used to determine the fundamental properties unambiguously.

In any case, a parametrization of the whole transport model is not needed to develop a useful version of the MacInnes equation for immediate application in P2D simulations. One only requires the conductivity κ and the junction coefficients
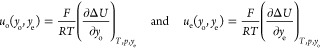
12which can be obtained directly from a functional correlation that establishes Δ*U*(*y*_o_,*y*_e_). Experimental concentration-cell data are used to develop such a correlation in the next section.

## 3 Data for EMC:EC:LiPF_6_ Ternaries

Concentration-cell measurements were performed following the method and apparatus described previously by Hou et al.^34^ Some minor additions were made to the prior procedure to account for the use of ternary solutions.

For all experiments, ternary EMC:EC:LiPF_6_ stock solutions were prepared within an argon glovebox (Inert Technologies) with vacuum-dried LiPF_6_ (99.99%, battery grade, Sigma-Aldrich), as well as EC (99%, Sigma-Aldrich) and EMC (99.9%, Sigma-Aldrich) solvents that were stored in the glovebox under 3 Å molecular sieves to ensure dryness. Target electrolyte compositions were formulated gravimetrically, by weighing out appropriate amounts of the pure components and a nominally 2 M LiPF_6_ in EMC stock solution (battery grade, Sigma-Aldrich). Note that densitometry was performed on the stock solution at 25.00 ± 0.02 °C and the density correlation reported by Wang et al.^6^ was inverted to establish composition of the EMC:LiPF_6_ precursor more precisely at 2.007 M (25 °C), corresponding to a temperature-independent salt mass fraction of 0.2462 in the stock solution.

Every concentration-cell measurement was performed within the glovebox, whose atmosphere was maintained at 25 °C by a thermostat. For each experiment, 5.0 mL samples of the reference and test solutions were pipetted simultaneously into a fritted (D-type, 10–16 μm) glass “H-cell”. Identical lithium-metal foil electrodes (99.9% Alfa Aesar) were used as reference electrodes. Electrodes were polished with a PTFE brush before being submerged into the electrolyte within the test and reference chambers of the H-cell. Liquid-junction potentials were recorded over a stabilization period of 30 min at 25 °C, and the steady-state open-circuit potential was measured with a potentiostat (PGSTAT302N, Metrohm).

**Figure 1:**
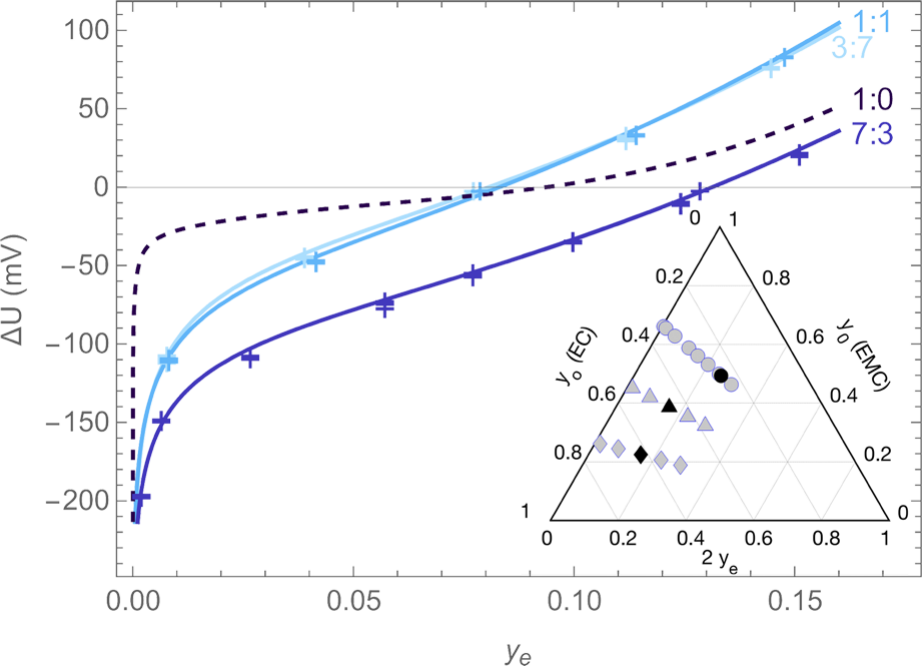
Liquid-junction potential measurements relative to the reference composition for 3:7, 1:1, and 7:3 solvent ratios (markers) are shown alongside best-fit curves using eq 13 (solid lines). The correlation measured by Wang et al.^6^ for the EMC:LiPF_6_ binary system (1:0 EMC:EC ratio) is shown for comparison (dashed line), referenced to the composition [*y*_o_, *y*_e_] = [0, 0.09379], at which the binary solution exhibits an ideal thermodynamic factor (*X*_ee_ = 0). Inset: Ternary plot showing solution compositions of test solutions (gray markers) and reference solutions (black markers) in concentration cells. Circles, triangles, and diamonds, respectively, indicate solutions prepared with EMC:EC ratios of 7:3, 1:1, and 3:7.

An initial set of liquid-junction-potential measurements was made using test solutions with compositions indicated by the gray markers shown on the ternary diagram in the inset of Figure 1. The raw experimental data used to generate this plot are presented in tabular form in the Supporting Information (Section S1). Liquid-junction potentials were first measured by varying salt content alone, using sets of reference and test solutions with equal EMC:EC solvent mass ratios of 7:3 (circular markers in the inset of Figure 1), 1:1 (triangles), and 3:7 (diamonds). The reference solutions were at compositions [*y*_o_, *y*_e_] = [0.2498, 0.1286], [*y*_o_, *y*_e_] = [0.4564, 0.07875], and [*y*_o_, *y*_e_] = [0.6212, 0.07723], respectively, and test-solution salt fractions were varied between *y*_e_ = 0 and *y*_e_ ≈ 0.15. At every EMC:EC ratio, the liquid-junction potential curves shown in Figure 1 tend toward large negative values (Δ*U* → – *∞*) as the salt fraction tends to zero (*y*_e_ → 0). This Nernstian dependence of concentration overpotential on salt concentration at low molarity has been widely observed.^6,10,39−42^ Note also that eq 10 suggests that *u*_e_ ∝ 1/*y*_e_ to leading order, which confirms a Nernstian dependence after the integration in eq 11. Data for Δ*U* at fixed solvent ratio were therefore fitted with nonlinear functions of the form
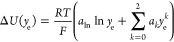
13correcting the expected logarithmic dependence with a second-order polynomial in *y*_e_. Parameters for fit eq 13 at the various solvent ratios are listed in Table 1, and were used to calculate the curves plotted alongside the experimental junction-potential data in Figure 1. The figure also presents the voltage correlation reported for the binary EMC:LiPF_6_ system by Wang et al.^6^

**Table 1:** Liquid-Junction Potential Fit Parameters for the Three Solvent Ratios

EMC:EC	*a*_ln_	*a*_0_	*a*_1_	*a*_2_
7:3	1.390	1.158	–8.955	164.7
1:1	1.491	3.007	–8.168	199.6
3:7	1.672	4.034	–14.57	207.6

**Figure 2:**
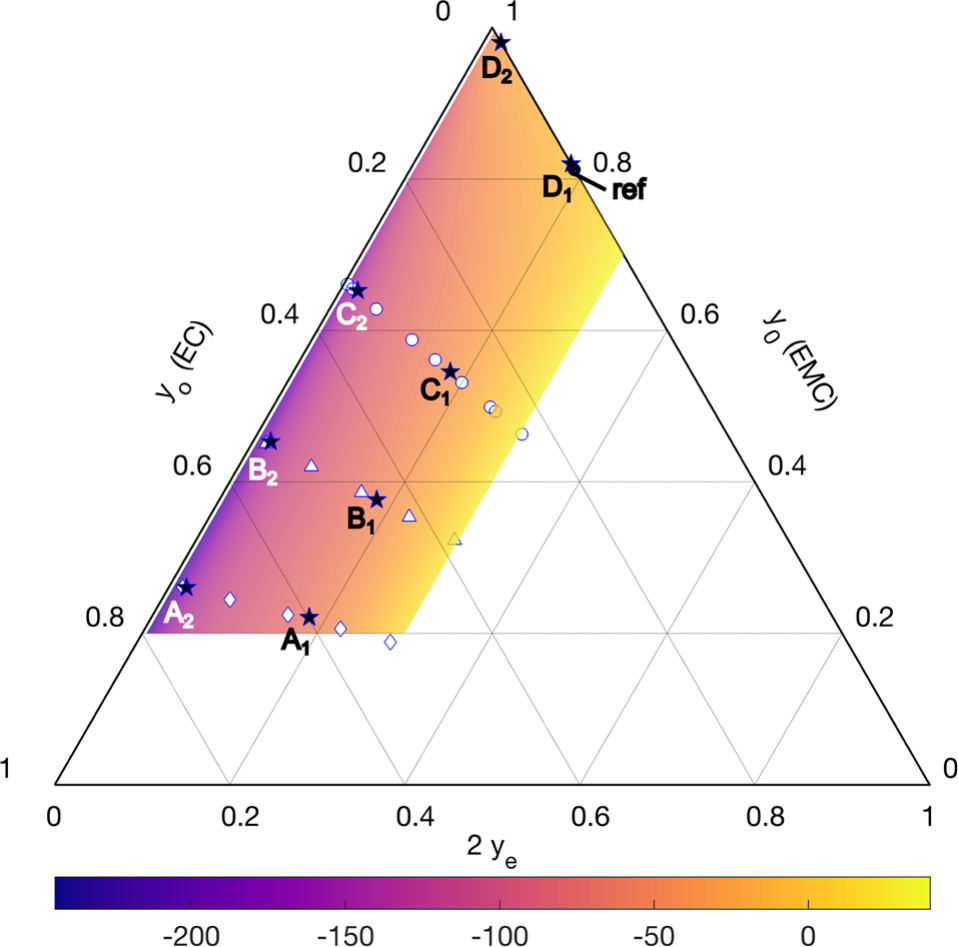
Ternary diagram showing all the compositions at which liquid-junction potentials were measured for EMC:EC:LiPF_6_ mixtures (markers). The color map shows the surface with the form of eq 17 that best fits the liquid-junction potential data (in units of mV) after they are shifted to a common reference solution with *y*_o_ = 0 and *y*_e_ = 0.09379 (composition “ref”).

To probe how junction potentials depend on cosolvent composition, another set of experiments was performed with fixed salt contents and varying solvent ratios. Figure 2 provides a ternary plot of the liquid-junction potential Δ*U*, with points labeled A_1_, B_1_, C_1_, D_1_, A_2_, B_2_, C_2_, and D_2_ marking the eight solution compositions used for these experiments. These compositions are tabulated in Table 2. Concentration cells were loaded with various pairwise combinations of the eight test solutions. Six voltage measurements were performed with each tested pair of compositions. Average junction-potential measurements for these combinations, as well as the standard error across the six measurements, are provided in Table 3.

**Table 2:** Particle Fractions Used for Constant-*y*_e_ Concentration-Cell Measurements

Position	*y*_o_	*y*_e_	Position	*y*_o_	*y*_e_
A_1_	0.5985	0.09017	A_2_	0.7191	0.01019
B_1_	0.4438	0.08995	B_2_	0.5265	0.01027
C_1_	0.2753	0.08974	C_2_	0.3273	0.01010
D_1_	0	0.09009	D_2_	0	0.01004

**Table 3:** Liquid-Junction Potential Measurements at Constant Salt Particle Fractions

Ref. | Test	Δ*U* (mV)	Ref. | Test	Δ*U* (mV)
A_1_ | B_1_	8.4 ± 0.5	A_2_ | B_2_	14.9 ± 0.1
A_1_ | C_1_	22.3 ± 0.2	A_2_ | C_2_	40.0 ± 0.8
A_1_ | D_1_	57 ± 3	A_2_ | D_2_	132.6 ± 0.8
B_1_ | C_1_	13.6 ± 0.3	B_2_ | C_2_	23.86 ± 0.06
B_1_ | D_1_	49.7 ± 0.6	B_2_ | D_2_	116.3 ± 0.7
C_1_ | D_1_	35.26 ± 0.09	C_2_ | D_2_	91 ± 1

The data with different solvent ratios were used to shift reference potentials as follows. First, the junction-potential measurements with varying cosolvent ratio in Table 3 were used to shift the 7:3, 1:1, and 3:7 fixed-ratio voltage curves to reflect composition D_1_ as a common reference state. The functions yielded by fit eq 13, with parameters from Table 1, were used to determine the shift constants, such that the respective curves crossed the experimental voltages Δ*U*(A_1_–D_1_), Δ*U*(B_1_–D_1_), and Δ*U*(C_1_–D_1_) when reaching compositions A_1_, B_1_, and C_1_, respectively. To impart more physical meaning to the reference state, the common reference composition was additionally shifted away from D_1_ to **y**^ref^ = [0, 0.09379], the point at which Wang et al. observed that *X*_ee_ = 0 for EMC:LiPF_6_.^52^ Wang et al. provide a correlation for *t*_+_^0^(*y*_e_) and a thermodynamic-factor correlation that can be used to calculate *X*_ee_(*y*_e_).^6^ Through the integral in eq 11, these produce a correlation for how the liquid-junction potential relative to **y**^ref^ along the no-EC edge of the ternary space, Δ*U*_1:0_, varies with respect to *y*_e_:
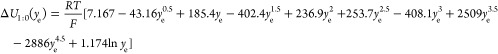
14Since point D_1_ lies along the no-EC edge of the ternary space, the junction potential between composition D_1_ and **y**^ref^ is given by Δ*U*_1:0_(D_1_). This additional constant was added to the results that had already been expressed relative to composition D_1_. Figure 2 presents the measured liquid-junction potential data across the ternary composition space relative to a common reference composition **y**^ref^ = [0, 0.09379]. These data are also given in tabular form in the Supporting Information, Section S2.

## 4 Junction-Potential Correlation for EMC:EC:LiPF_6_

Composition dependences of the junction coefficients *u*_o_ and *u*_e_ must be specified to compute concentration overpotentials with the transport model. Since these derive from partial derivatives of the state function Δ*U*(*y*_o_,*y*_e_) that quantifies liquid-junction potential with respect to composition, cf. eq 12, a meaningful and consistent best fit of the experimental data is needed. Methods for fitting state functions from classical chemical thermodynamics^43^ were consequently adopted to fit the surface Δ*U*(*y*_o_,*y*_e_).

The liquid-junction potential along the no-EC edge of the ternary composition space—where *y*_e_ varies but *y*_o_ = 0—is known very accurately from the data of Wang et al. Thus, the functional form of the correlation for Δ*U*(*y*_o_,*y*_e_) was designed such that
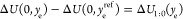
15with Δ*U*_1:0_(*y*_e_) given relative to *y*_e_^ref^ = 0.09379 by eq 14. To fit data, we imagine that the edge of the ternary space where *y*_o_ = 1–2*y*_e_ (i.e., *y*_0_ = 0) can also be modeled by a function dependent only on *y*_e_,
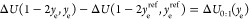
16in which the function Δ*U*_0:1_(*y*_e_) quantifies how liquid-junction potential would be expected to vary with salt content in pure EC. It should be borne in mind that the EC:LiPF_6_ edge of the EMC:EC:LiPF_6_ composition space is not directly experimentally accessible because EC is not a liquid at room temperature. This assumed functionality nevertheless captures the idea that the liquid-junction potential along this edge of the composition space is expected to behave similarly to a binary electrolytic solution.

To accommodate the binary property correlations described by eqs 15 and 16 in a consistent way, the liquid-junction potential data were fit using a function with the general structure
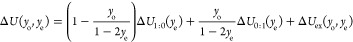
17in which the excess liquid-junction potential Δ*U*_ex_ accounts for deviation from the composition-weighted average of Δ*U*_1:0_ and Δ*U*_0:1_ at interior points within the ternary composition space. The prefactors weighting the latter two functions enforce the properties demanded by eqs 15 and 16; their forms derive from the facts that physical salt fractions lie within the range 0 ≤ *y*_e_ ≤ 1/2, and EC contents, within 0 ≤ *y*_o_ ≤ 1–2*y*_e_. Choices for fitting the excess junction potential in eq 17 must also be constrained such that Δ*U*_ex_(0,*y*_e_) = 0 and Δ*U*_ex_(1–2*y*_e_,*y*_e_) = 0.

**Figure 3:**
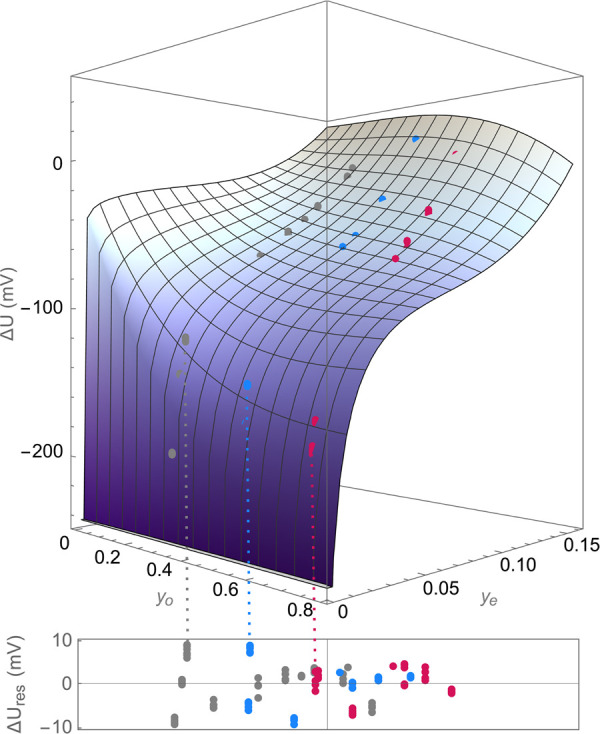
(Top) Liquid-junction potential surface Δ*U*(*y*_o_,*y*_e_) yielded by fitting eq 17 to experimental data (marks). Data sets corresponding to different EMC:EC cosolvent ratios are distinguished with gray (7:3), blue (1:1), and red (3:7) markers. (Bottom) Scatter plot showing the residual voltage difference Δ*U*_res_ between each experimental data point and the best-fit surface.

Along the no-EMC edge of the composition space, the liquid-junction potential was described with a function in the form of eq 13:
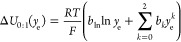
18involving four constant parameters *b*_*k*_. Inspired by the three-suffix Margules correction for ternary solutions from thermodynamics, the excess liquid-junction potential across the ternary space was assumed to take the form
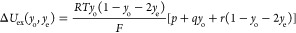
19dependent on constants *p*, *q*, and *r*. To establish the correlation for Δ*U*(*y*_o_,*y*_e_), the measured liquid-junction potential data were expressed relative to a common reference composition **y**^ref^ = [0, 0.09379] using the process discussed earlier. Then, the parameters in eqs 18 and 19 were estimated by directly fitting eq 17 to the shifted liquid-junction-potential data (tabulated in the Supporting Information, section S2) with the method of least-squares. This yielded the parameter set listed in Table 4, which produces the best-fit surface shown in Figure 3. A satisfactory fit is achieved by these parameters, as illustrated by the residual voltages (the difference between the measured liquid-junction potentials and the fit); Figure 3 shows that these are scattered densely near 0 mV and deviate by at most 10 mV. Bear in mind that the use of this correlation for Δ*U*(*y*_o_,*y*_e_) should be restricted to the range 0 < *y*_o_ < 0.75 and 0.002 < *y*_e_ < 0.15 to avoid extrapolation.

**Table 4:** Fit Parameters for the Liquid-Junction Potential Correlation in eqs 17, 18, and 19

*b*_ln_	*b*_0_	*b*_1_	*b*_2_
3.024	8.233	–88.12	477.9

*p*	*q*	*r*
32.20	–37.99	–44.80

## 5 Results and Discussion

Figures 2 and 3 present the variation of liquid-junction potential with respect to ternary composition in two different ways. The most striking feature of the junction-potential surface, perhaps clearest from Figure 2, is that significant voltage differences are registered between different cosolvent fractions at constant salt content *y*_e_. Appreciable electrical potential, induced by a difference in the electrochemical potential of dissolved lithium cations, arises across a junction between two reservoirs of the same salt particle fraction but different EMC:EC ratios. Since the variation of Δ*U* with the cosolvent ratio is significant, especially at low salt particle fractions, the electrical aspect of the single-solvent approximation does not appear to hold for the EMC:EC:LiPF_6_ electrolyte system.

For the observed variation of junction potential with cosolvent ratio to be practically important in batteries, however, it is also necessary that the cosolvent distribution be changed by applied current. Interfacial reactions with lithium can induce solvent transport in two main ways. Faradaic convection occurs when the consumption or production of Li^+^ at solution boundaries impermeable to solvent drives bulk flow,^44^ and is described by the convective  terms in flux laws 8. Electroosmotic solvent transport occurs when current-carrying ions exert more diffusional drag on one solvent (EC, say) than the other (EMC) as they move. This phenomenon is quantified by the migration coefficient Ξ̃.

Strong evidence for the solvent dependence of ion energetics exists in the literature. Raman spectroscopy by Jeong et al.^45^ confirmed preferential accumulation of EC over dimethyl carbonate (DMC) and diethyl carbonate (DEC)—both structurally similar to EMC—in the Li^+^ solvation shell, although the measurements were performed at a fixed salt concentration with a single cosolvent ratio (1:1 by volume). Using the same technique, a more comprehensive study by Uchida and Kiyobayashi^46^ indicated that EC is about twice as likely to appear than DMC in the Li^+^ solvation shell in low-EC mixtures, a preferential effect that decreases as the EC concentration is raised. More directly, Von Wald Cresce et al.,^47^ through electrospray ionization mass spectrometry, found that EC is much more strongly favored in the solvation shell of Li^+^ over EMC and again, this was especially pronounced in the lower range of EC particle fraction.

Ternary junction-potential correlations make it possible to compute how given swings in salt content and cosolvent composition will contribute to a battery’s OCV. Variations of salt concentration produce much higher overpotential than variations of solvent concentration, but the latter effect is still appreciable. Taylor expansion of Δ*U* around the baseline composition of 1:1 EMC:EC and 1 M LiPF_6_ at 25 °C (i.e., [*y*_o_,*y*_e_] = [0.4615,0.0740], or [*c*_0_,*c*_o_,*c*_e_] = [5.27 M,6.24 M,1.00 M]) studied by Wang et al.^31^ shows that salt concentration polarization produces 53.4 mVM^–1^ of overpotential, whereas EC polarization produces −5.85 mVM^–1^. Although the former effect is much larger than the latter, both can be important if solvent migration is significant.

Prior experiments suggest that solvent concentration polarization is larger than salt polarization for this electrolyte system. They also show that polarization of salt molarity opposes the polarization of EC molarity, so the overpotentials that arise from molarity changes of salt and EC enforce each other, rather than canceling each other out. Wang et al.^31^ used their Hittorf results to show that the current pulses they applied induced a swing in the EMC:EC mass ratio at the cathode surface (where lithium ions are reduced) from 1:1 to 1:1.6—a rise of 2.0 M in the EC concentration relative to the bulk—accompanied by a LiPF_6_ concentration drop of 0.8 M, from 1.0 to 0.2 M; at the anode surface, EMC:EC swung from 1:1 to 1:0.6, falling by 2.0 M as the salt concentration rose by 0.9 M, from 1.0 to 1.9 M. Both electrodes sustained changes in the EC concentration that were about twice that of the salt. According to the Δ*U* correlation established here, the total concentration overpotential relative to the bulk at the cathode in Wang’s experiment was −75.3 mV, with −6.1 mV (8%) contributed by EC polarization; at the anode the overpotential was 74.8 mV, with 7.3 mV (10%) coming from EC.^53^ The portion of these overpotentials due to the solvent distribution is neglected by almost all state-of-the art P2D models. Based on the size of observed current-induced changes in the cosolvent ratio, however, it may be important to account for how solvent distributions affect the cell voltage when modeling battery performance at high current.

### 5.1 Thermodynamic Consistency of Liquid-Junction Potentials

One should be aware that all of the analysis in this paper assumes that the electroneutral Onsager–Stefan–Maxwell theory reasonably models this electrolyte system. Of particular importance is the assumption that the voltage Φ involved in the MacInnes equation, eq 9, is a path-independent state function at open circuit, which was invoked when deriving eq 11 to express Δ*U* as a thermodynamic path integral. The utility of the correlation for Δ*U*(*y*_o_,*y*_e_) is predicated on its path independence in the composition space.

For a measurement of Δ*U*(*y*_o_,*y*_e_) to be path independent, any energy dissipated during the measurement process—in this case, concentration-cell experiments—must be negligible. The experiments reported here broadly indicated that the voltage drop across the concentration cells changed minimally over time, suggesting quasi-static behavior. This does not ensure reversibility of the process being measured, however.

**Figure 4:**
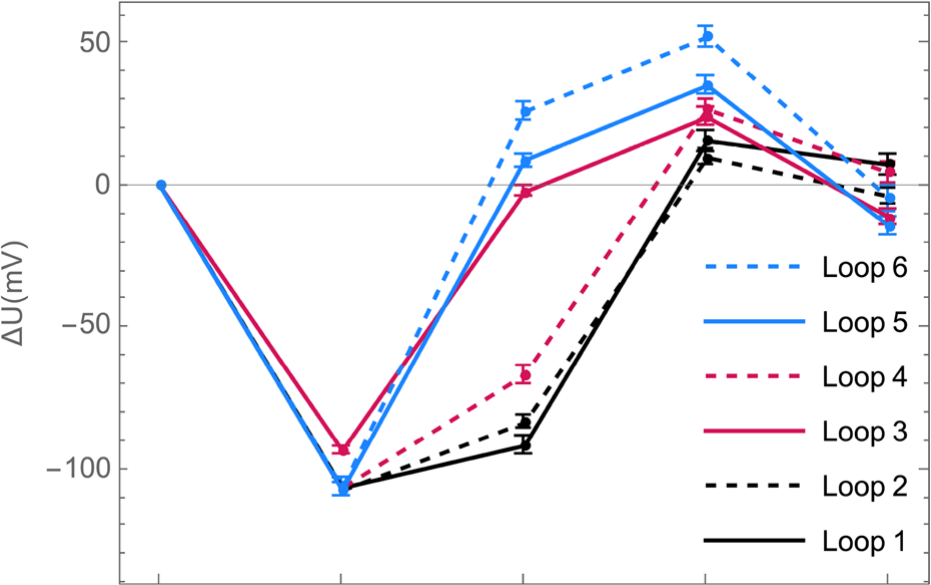
Net liquid-junction potential around the six loops in ternary composition space described in Table 5.

The reversibility aspect of the junction potential was tested by examining concentration-cell experiments that traverse closed paths in the composition space, around which the net voltage change should be zero if irreversible losses are negligible. Six closed loops were drawn from concentration-cell experiments between the composition points listed in Table 2. These data were combined with the functional forms of the liquid-junction potentials for each fixed solvent ratio provided in eq 13, with the necessary parameters from Table 1. The accumulated voltage (∑Δ*U*) around various loops in the composition space is provided in Table 5, as well as the total voltage traversed, (∑|Δ*U*|). The voltage drops around various loops listed in the table are depicted graphically on Figure 4.

**Table 5:** Liquid-Junction Potentials Measured Experimentally around Six Closed Loops in the Composition Space

	Path	∑Δ*U* (mV)	∑|Δ*U*| (mV)	|∑Δ*U*|/∑|Δ*U*|
Loop 1	A_1_-A_2_-B_2_-B_1_-A_1_	7	237	0.030
Loop 2	B_1_-B_2_-C_2_-C_1_-B_1_	–4	238	0.017
Loop 3	C_1_-C_2_-D_2_-D_1_-C_1_	–11	246	0.045
Loop 4	A_1_-A_2_-C_2_-C_1_-A_1_	5	262	0.019
Loop 5	B_1_-B_2_-D_2_-D_1_-B_1_	–14	300	0.047
Loop 6	A_1_-A_2_-D_2_-D_1_-A_1_	–5	322	0.016
Average		–4	268	0.015

All the loops closed within an average of 1.5% of the total voltage traversed, and both voltage gains and losses were encountered. Notably, the four legs that make up each loop traverse hundreds of millivolts in total, see Figure 4. Around all six loops the mismatch voltages are densely distributed and take both positive and negative values. The biggest misalignment comes from Loop 5, whose mismatch was −14 mV, or 4.7% of the total voltage traversed (∼300 mV). This is about double the size of the residuals shown in Figure 3, and thus not far outside the range expected based on experimental error. Liquid-junction potentials appear to be reversible enough to justify the use of experimental correlations derived from concentration cells in the transport model.

### 5.2 Junction Coefficients

The liquid-junction potential parametrization allows direct computation of the junction coefficients *u*_o_ and *u*_e_ with the formulas from eq 12.

**Figure 5:**
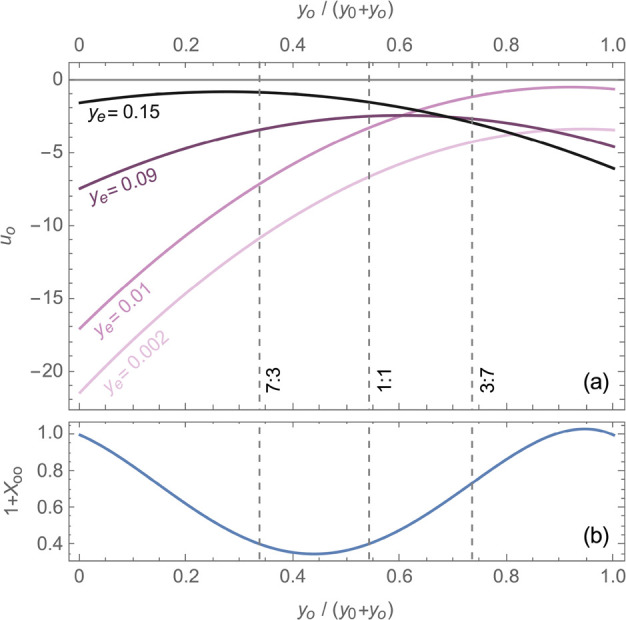
Plots against the EC mole fraction in neat cosolvent of (a) the junction coefficient *u*_o_ for four salt fractions *y*_e_ and (b) the thermodynamic factor 1 + *X*_oo_ of a salt-free EMC:EC binary mixture derived from the correlation of Ding.^48^

**Figure 6:**
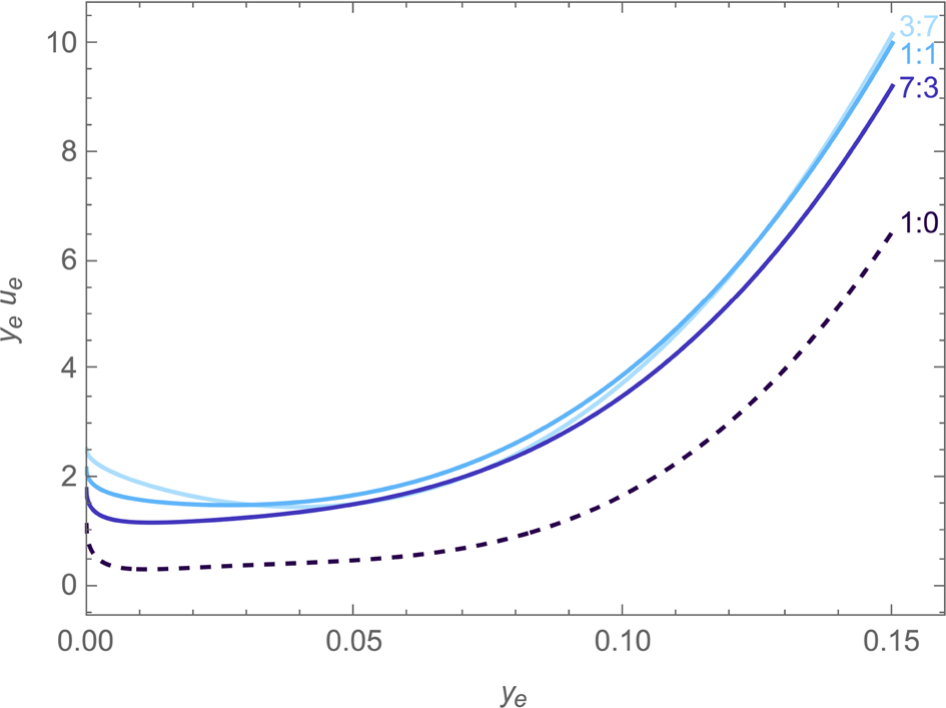
Junction coefficient *u*_e_ weighted by the salt particle fraction *y*_e_ at EMC:EC mole ratios of 3:7, 1:1, 7:3, and 1:0.

Figure 5(a) shows *u*_o_(*y*_o_, *y*_e_) for *y*_e_ = 0.002, 0.01, 0.09, and 0.15. The first and last of these values correspond to the boundaries of the experimental composition range; 0.01 and 0.09 correspond to *y*_e_ along the A_2_-B_2_-C_2_-D_2_ and A_1_-B_1_-C_1_-D_1_ paths (see Figure 2), respectively. Figure 6 presents the values of *y*_e_*u*_e_(*y*_o_, *y*_e_), a more smoothly varying combination that cancels out the leading-order 1/*y*_e_ dependence expected from eq 10.^54^ This quantity is shown for the three experimentally tested cosolvent ratios, as well as the results Wang et al. presented for the 1:0 solvent mix. The junction coefficients vary with both salt and EC content, but the effect of salt content is stronger, especially for *u*_o_ when salt is dilute. This is not surprising given the expected Nernstian behavior of concentration overpotential in the dilute regime. Significantly, in the moderately concentrated region where Li-ion batteries usually operate (NB: *y*_e_ = 0.08902 for 1 M LiPF_6_ in EMC), *u*_o_ is sizable enough that both junction coefficients are needed to describe the voltage accurately. This again supports the argument that the single-solvent approximation may not suffice for high-fidelity battery models.

It is possible to draw more detailed conclusions about EC migration by inspecting *u*_o_ from Figure 5(a) in relation to eqs 10. As the salt in solution becomes more and more dilute, *u*_o_ appears to decrease substantially. This trend may either owe to composition dependence of the thermodynamic factors *X*_oo_ and *X*_ee_, or to the composition dependences of the electro-osmotic coefficient and transference number. Ding studied vapor–liquid equilibrium of EMC:EC binary mixtures,^48^ providing correlations that can be used to obtain the composition dependence of activity coefficients, and consequently *X*_oo_, in the absence of salt. The chemical-potential correlations by Ding^48^ at 25 °C predict that *X*_oo_(*y*_o_,0) varies as
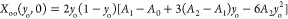
20where *A*_0_ = 1.0471, *A*_1_ = 0.42506, and *A*_2_ = −0.85568. As shown in the lower panel of Figure 5(b), the factor (1 + *X*_oo_), which multiplies Ξ̃ in eq 10, ranges between ∼0.4 and ∼1 across the cosolvent composition range, with a minimum near *y*_o_ = 0.4. Whereas *u*_o_ varies by a factor of 10 or more across the cosolvent composition range, (1 + *X*_oo_) varies by a factor nearer 2. Furthermore, (1 + *X*_oo_) decreases in most of the range where *u*_o_ increases. Thus, cosolvent thermodynamics at high dilution does not likely control either the composition dependence of *u*_o_ or its large magnitude.

In a prior model derived for lithium–oxygen-battery electrolytes,^49^ Monroe provided results which show that Ξ̃ can be written in terms of fundamental transport properties and composition variables as
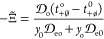
21where  and  are the respective thermodynamic diffusivities of salt in EC and EMC, dependent only on Stefan–Maxwell coefficients, and *t*_+0̷_^o^ and  are respectively the (Hittorf) cation transference numbers relative to pure EC and pure EMC. The negative value of *u*_o_ across the composition range is commensurate with a conclusion that lithium transference is greater in EMC than in EC. Past studies have shown that up to moderate dilution, Li^+^ has a higher Hittorf transference number in EMC^12^ than PC,^34^ the latter being a cyclic carbonate similar to EC. The large magnitudes of *u*_o_ across the composition range suggest that the thermodynamic diffusivities of salt in both solvents ( and ) are substantially lower than EC’s component diffusivity in the solution . Better understanding of the *u*_o_ measurements could be gained from future composition-dependent characterization of diffusion and transference across the ternary space.

It remains possible that the excess thermodynamic factor *X*_oo_ is much larger when salt is present than in its absence. Assuming that contributions by *X*_eo_ are relatively small, one would expect *u*_o_ to be proportional to (1 + *X*_oo_). Obviously, *X*_oo_ in the ternary mixture depends on two composition variables, so it almost certainly differs from *X*_oo_ in the EMC:EC binary. Still, the activity coefficients of species in solution tend to vary most strongly as the species’ own concentrations change.

An estimate for the migration coefficient of 1 M LiPF_6_ in 1:1 mass ratio EMC:EC is available from Wang et al., who processed Hittorf measurements for the ternary system with a Gaussian process regression technique.^31^ The quantity they report, Ξ ( in the present notation^55^), suggests that Ξ̃ = 3.0 ± 0.2. At this composition, assuming that *X*_oo_ follows Ding’s correlation (eq 20) and *X*_eo_ ≈ 0, our *u*_o_ measurement produces Ξ̃ = 3.2 through eq 10. The fair agreement between these values suggests that it is reasonable to employ thermodynamic simplifications to estimate Ξ̃ values from junction-coefficient measurements. Notably, junction coefficients are far easier to measure across the composition space than Hittorf transference numbers, so this route for property estimation has substantial practical utility.

Last, the observed trends in *u*_o_ also admit the possibility that *X*_eo_ has a stronger composition dependence than expected. Although the Maxwell relation in eq 4 suggests that *X*_eo_ = *O*(*y*_e_) or higher, any dependence of order , where 0 < α < 1, could induce the observed trend. It is possible that a regime exists below the salt concentrations studied in which *X*_eo_ varies in this way before returning to the constant value thermodynamic stability demands at infinite dilution. More extensive thermodynamic experiments probing vapor–liquid equilibrium in the ternary space will be needed to determine the validity of any hypotheses relevant to solution thermodynamics for the EMC:EC:LiPF_6_ electrolyte system.

## 6 Conclusion

Liquid-junction potentials in concentration cells containing EMC:EC:LiPF_6_ solutions vary significantly with respect to the cosolvent ratio at fixed salt content. In combination with the significant electro-osmotic coefficient of EC in EMC, these observations contradict basic assumptions that underpin the single-solvent approximation on which most physics-based models of lithium-ion batteries rely. An extended transport model based in Onsager–Stefan–Maxwell concentrated-solution theory was developed to rationalize liquid-junction potential measurements across the ternary composition space in a thermodynamically consistent way. Junction coefficients, parameters that model concentration dependent overpotentials associated with individual solution components, were identified in the modified form of Ohm’s law, and can be calculated directly from liquid-junction-potential correlations. Solutions with EMC:EC ratios of 3:7, 1:1, and 7:3 at a wide range of salt concentrations were used to quantify liquid-junction potential, and junctions with fixed salt content but varying solvent content were used to express all of the measured potentials relative to a common reference state (an EMC:LiPF_6_ binary solution at the concentration where its thermodynamic factor is ideal). Analysis of the potential measurements justified the assertion that liquid-junction potential can be treated as a state function. A ternary liquid-junction-potential correlation was created by fitting the commonly referenced experimental data with functional forms inspired by chemical thermodynamics. This correlation, which is the major result of the present work, was used to probe the magnitudes and composition-dependent behavior of the junction coefficients of EC and LiPF_6_. Given that the single-solvent approximation does not appear to hold for the common EMC:EC:LiPF_6_ electrolyte formulation, future experimental and theoretical work will be needed to understand multicomponent electrochemical transport in this and other cosolvent lithium-ion electrolytes.

## A. Partial Molar Volume Measurement

Newman shows how the partial molar volumes of constituents within an *n*-component solution can be extracted from measurements of solution density with respect to *n* – 1 component molarities.^10^ Assuming the functionality of ρ(*c*_o_,*c*_e_) at constant temperature and pressure across the EMC:EC:LiPF_6_ ternary composition space is known, Newman’s general formula shows that

22

in which , , and  stand for the molar masses of EMC, EC, and LiPF_6_, respectively. Wang et al. reported densities for ternary EMC:EC:LiPF_6_ solutions with different component mass fractions.^31^ Section S3 of the Supporting Information recasts this data in terms of component molarities to facilitate the estimation of partial molar volumes.

## B. Fickian diffusivity

The diffusivities that appear in eq 8 can be defined compactly in terms of previously introduced properties by leveraging matrix algebra. Let

23

define a thermodynamic diffusivity matrix , a dimensionless thermodynamic matrix **X**, and a dimensionless molarity conversion matrix **C**. The four Fickian diffusivities *D*_oo_^0^, *D*_oe_^0^, *D*_eo_^0^, and *D*_ee_^0^ are then defined by the matrix product

24

Matrices **X** and **C** are both generally invertible, so this relationship can be manipulated to express any one of the matrices as a product of the others (or their inverses). The matrix  is symmetric, and the entries of **X** are constrained by Maxwell relation 4. Neither **X** nor **C** is generally diagonal or symmetric. Formally, the ideal-solution approximation assumes that *X*_*ij*_ = 0 for all *i* and *j*, in which case thermodynamic matrix **X** becomes diagonal and constant; the dilute-solution approximation would additionally take *c*_o_ ≪ *c*_0_ and *c*_e_ ≪ *c*_0_, such that molarity conversion matrix **C** becomes the identity matrix.

## Supporting Information Available

Tabulated raw junction-potential data (section S1); tabulated junction-potential data shifted to a common reference (section S2); density and total concentration data expressed with respect to component mass fractions, component molarities, and component particle fractions (section S3) (PDF)

## References

[ref1] Beard K. W. Linden’s Handbook of Batteries, 5th ed.; 2019.

[ref2] Xu K. Nonaqueous Liquid Electrolytes for Lithium-Based Rechargeable Batteries. Chem. Rev. 2004, 104, 4303–4318. 10.1021/cr030203g. 15669157

[ref3] Xu K. Electrolytes and Interphases in Li-Ion Batteries and Beyond. Chem. Rev. 2014, 114, 11503–11618. 10.1021/cr500003w. 25351820

[ref4] Landesfeind J.; Ehrl A.; Graf M.; Wall W. A.; Gasteiger H. A. Direct Electrochemical Determination of Thermodynamic Factors in Aprotic Binary Electrolytes. J. Electrochem. Soc. 2016, 163, A1254–A1264. 10.1149/2.0651607jes.

[ref5] Landesfeind J.; Gasteiger H. A. Temperature and Concentration Dependence of the Ionic Transport Properties of Lithium-Ion Battery Electrolytes. J. Electrochem. Soc. 2019, 166, A3079–A3097. 10.1149/2.0571912jes.

[ref6] Wang A. A.; Hou T.; Karanjavala M.; Monroe C. W. Shifting-reference concentration cells to refine composition-dependent transport characterization of binary lithium-ion electrolytes. Electrochim. Acta 2020, 358, 136688 10.1016/j.electacta.2020.136688.

[ref7] Zhang S. S.; Jow T. R.; Amine K.; Henriksen G. L. LiPF_6_–EC–EMC electrolyte for Li-ion battery. J. Power Sources 2002, 107, 18–23. 10.1016/S0378-7753(01)00968-5.

[ref8] Zhang L.; Chai L.; Zhang L.; Shen M.; Zhang X.; Battaglia V. S.; Stephenson T.; Zheng H. Synergistic effect between lithium bis(fluorosulfonyl)imide (LiFSI) and lithium bis-oxalato borate (LiBOB) salts in LiPF_6_-based electrolyte for high-performance Li-ion batteries. Electrochim. Acta 2014, 127, 39–44. 10.1016/j.electacta.2014.02.008.

[ref9] Bizeray A. M.; Howey D. A.; Monroe C. W. Resolving a discrepancy in diffusion potentials, with a case study for Li-Ion batteries. J. Electrochem. Soc. 2016, 163, E223–E229. 10.1149/2.0451608jes.

[ref10] Newman J.; Thomas-Alyea K. E. Electrochemical systems, 3rd ed.; 2004.

[ref11] Lightfoot E. N.; Cussler E. L.; Rettig R. L. Applicability of the Stefan–Maxwell equations to multicomponent diffusion in liquids. AIChE J. 1962, 8, 708–710. 10.1002/aic.690080530.

[ref12] Wang A. A.; Gunnarsdottir A. B.; Fawdon J.; Pasta M.; Grey C. P.; Monroe C. W. Potentiometric MRI of a Superconcentrated Lithium Electrolyte: Testing the Irreversible Thermodynamics Approach. ACS Energy Letters 2021, 6, 3086–3095. 10.1021/acsenergylett.1c01213. 34541321 PMC8438662

[ref13] Doyle M.; Fuller T. F.; Newman J. Modeling of Galvanostatic Charge and Discharge of the Lithium/Polymer/Insertion Cell. J. Electrochem. Soc. 1993, 140, 1526–1533. 10.1149/1.2221597.

[ref14] Brosa Planella F.; et al. A continuum of physics-based lithium-ion battery models reviewed. Progress in Energy 2022, 4, 042003 10.1088/2516-1083/ac7d31.

[ref15] Andersson M.; Streb M.; Ko J.; Löfqvist Klass V.; Klett M.; Ekström H.; Johansson M.; Lindbergh G. Parametrization of physics-based battery models from input–output data: A review of methodology and current research. J. Power Sources 2022, 521, 230859 10.1016/j.jpowsour.2021.230859.

[ref16] Wang A.; O’Kane S.; Brosa Planella F.; Le Houx J.; O’Regan K.; Zyskin M.; Edge J.; Monroe C.; Cooper S.; Howey D.; Kendrick E.; Foster J. Review of parameterisation and a novel database (LiionDB) for continuum Li-ion battery models. Progress in Energy 2022, 4, 032004 10.1088/2516-1083/ac692c.

[ref17] Dees D. W.; Kawauchi S.; Abraham D. P.; Prakash J. Analysis of the Galvanostatic Intermittent Titration Technique (GITT) as applied to a lithium-ion porous electrode. J. Power Sources 2009, 189, 263–268. 10.1016/j.jpowsour.2008.09.045.

[ref18] Krachkovskiy S. A.; Foster J. M.; Bazak J. D.; Balcom B. J.; Goward G. R. Operando Mapping of Li Concentration Profiles and Phase Transformations in Graphite Electrodes by Magnetic Resonance Imaging and Nuclear Magnetic Resonance Spectroscopy. J. Phys. Chem. C 2018, 122, 21784–21791. 10.1021/acs.jpcc.8b06563.

[ref19] Castle M.; Richardson G.; Foster J. M. Understanding rapid charge and discharge in nano-structured lithium iron phosphate cathodes. European Journal of Applied Mathematics 2022, 33, 328–368. 10.1017/S0956792521000036.

[ref20] Valøen L. O.; Reimers J. N. Transport Properties of LiPF_6_-Based Li-Ion Battery Electrolytes. J. Electrochem. Soc. 2005, 152, A882–A891. 10.1149/1.1872737.

[ref21] Stewart S.; Newman J. Measuring the Salt Activity Coefficient in Lithium-Battery Electrolytes. J. Electrochem. Soc. 2008, 155, A458–A463. 10.1149/1.2904526.

[ref22] Nyman A.; Behm M.; Lindbergh G. Electrochemical characterisation and modelling of the mass transport phenomena in LiPF_6_–EC–EMC electrolyte. Electrochim. Acta 2008, 53, 6356–6365. 10.1016/j.electacta.2008.04.023.

[ref23] Lundgren H.; Behm M.; Lindbergh G. Electrochemical Characterization and Temperature Dependency of Mass-Transport Properties of LiPF_6_ in EC:DEC. J. Electrochem. Soc. 2015, 162, A413–A420. 10.1149/2.0641503jes.

[ref24] Bergstrom H. K.; Fong K. D.; McCloskey B. D. Interfacial Effects on Transport Coefficient Measurements in Li-ion Battery Electrolytes. J. Electrochem. Soc. 2021, 168, 060543 10.1149/1945-7111/ac0994.

[ref25] Farkhondeh M.; Pritzker M.; Delacourt C.; Liu S. S.-W.; Fowler M. Method of the Four-Electrode Electrochemical Cell for the Characterization of Concentrated Binary Electrolytes: Theory and Application. J. Phys. Chem. C 2017, 121, 4112–4129. 10.1021/acs.jpcc.6b11501.

[ref26] Mistry A.; Grundy L. S.; Halat D. M.; Newman J.; Balsara N. P.; Srinivasan V. Effect of Solvent Motion on Ion Transport in Electrolytes. J. Electrochem. Soc. 2022, 169, 040524 10.1149/1945-7111/ac6329.

[ref27] Steinrück H.-G.; et al. Concentration and velocity profiles in a polymeric lithium-ion battery electrolyte. Energy Environ. Sci. 2020, 13, 4312–4321. 10.1039/D0EE02193H.

[ref28] Schmidt F.; Schönhoff M. Solvate Cation Migration and Ion Correlations in Solvate Ionic Liquids. J. Phys. Chem. B 2020, 124, 1245–1252. 10.1021/acs.jpcb.9b11330. 31990553

[ref29] Halat D. M.; Fang C.; Hickson D.; Mistry A.; Reimer J. A.; Balsara N. P.; Wang R. Electric-Field-Induced Spatially Dynamic Heterogeneity of Solvent Motion and Cation Transference in Electrolytes. Phys. Rev. Lett. 2022, 128, 198002 10.1103/PhysRevLett.128.198002. 35622024

[ref30] Mistry A.; Srinivasan V.; Steinrück H.-G. Characterizing Ion Transport in Electrolytes via Concentration and Velocity Profiles. Adv. Energy Mater. 2023, 13, 2203690 10.1002/aenm.202203690.

[ref31] Wang A. A.; Greenbank S.; Li G.; Howey D. A.; Monroe C. W. Current-driven solvent segregation in lithium-ion electrolytes. Cell Reports Physical Science 2022, 3, 101047 10.1016/j.xcrp.2022.101047.

[ref32] Ma Y.; Doyle M.; Fuller T. F.; Doeff M. M.; De Jonghe L. C.; Newman J. The Measurement of a Complete Set of Transport Properties for a Concentrated Solid Polymer Electrolyte Solution. J. Electrochem. Soc. 1995, 142, 1859–1868. 10.1149/1.2044206.

[ref33] Lundgren H.; Scheers J.; Behm M.; Lindbergh G. Characterization of the Mass-Transport Phenomena in a Superconcentrated LiTFSI:Acetonitrile Electrolyte. J. Electrochem. Soc. 2015, 162, A1334–A1340. 10.1149/2.0961507jes.

[ref34] Hou T.; Monroe C. W. Composition-dependent thermodynamic and mass-transport characterization of lithium hexafluorophosphate in propylene carbonate. Electrochim. Acta 2020, 332, 135085 10.1016/j.electacta.2019.135085.

[ref35] Van-Brunt A.; Farrell P.; Monroe C. Structural electroneutrality in Onsager–Stefan–Maxwell transport with charged species. Electrochim. Acta 2023, 441, 141769 10.1016/j.electacta.2022.141769.

[ref36] Monroe C. W.; Wheeler D. R.; Newman J. Nonequilibrium linear response theory: Application to Onsager-Stefan-Maxwell diffusion. Ind. Eng. Chem. Res. 2015, 54, 4460–4467. 10.1021/ie503875c.

[ref37] Goyal P.; Monroe C. W. Thermodynamic factors for locally non-neutral, concentrated electrolytic fluids. Electrochim. Acta 2021, 371, 137638 10.1016/j.electacta.2020.137638.

[ref38] Mistry A.; Srinivasan V. Do we need an accurate understanding of transport in electrolytes?. Joule 2021, 5, 2773–2776. 10.1016/j.joule.2021.10.007.

[ref39] Taylor P. B. Electromotive Force of the Cell with Transference and Theory of Interdiffusion of Electrolytes. J. Phys. Chem. 1927, 31, 1478–1500. 10.1021/j150280a002.

[ref40] Guggenheim E. A. The conceptions of electrical potential difference between two phases and the individual activities of ions. J. Phys. Chem. 1929, 33, 842–849. 10.1021/j150300a003.

[ref41] Hartley G. XLI. Theory of the velocity of diffusion of strong electrolytes in dilute solution. London, Edinburgh, and Dublin Philosophical Magazine and Journal of Science 1931, 12, 473–488. 10.1080/14786443109461823.

[ref42] Guggenheim E. A. Thermodynamics: An advanced treatment for chemists and physicists, 3rd ed.; Series in physics; North-Holland Pub. Co. ; Interscience Publishers: Amsterdam: New York, 1957.

[ref43] Newman J.; Battaglia V. The Newman Lectures on Thermodynamics; Jenny Stanford Publishing, 2019; p 328.

[ref44] Liu J.; Monroe C. W. Solute-volume effects in electrolyte transport. Electrochim. Acta 2014, 135, 447–460. 10.1016/j.electacta.2014.05.009.

[ref45] Jeong S.-K.; Inaba M.; Iriyama Y.; Abe T.; Ogumi Z. Surface film formation on a graphite negative electrode in lithium-ion batteries: AFM study on the effects of co-solvents in ethylene carbonate-based solutions. Electrochim. Acta 2002, 47, 1975–1982. 10.1016/S0013-4686(02)00099-3.

[ref46] Uchida S.; Kiyobayashi T. How does the solvent composition influence the transport properties of electrolyte solutions? LiPF_6_ and LiFSA in EC and DMC binary solvent. Phys. Chem. Chem. Phys. 2021, 23, 10875–10887. 10.1039/D1CP00967B. 33908519

[ref47] Von Wald Cresce A.; Borodin O.; Xu K. Correlating Li^+^ Solvation Sheath Structure with Interphasial Chemistry on Graphite. J. Phys. Chem. C 2012, 116, 26111–26117. 10.1021/jp303610t.

[ref48] Ding M. S. Excess Gibbs Energy of Mixing for Organic Carbonates from Fitting of Their Binary Phase Diagrams with Nonideal Solution Models. J. Solution Chem. 2005, 34, 343–359. 10.1007/s10953-005-3054-z.

[ref49] Monroe C. W. Does oxygen transport affect the cell voltages of metal/air batteries?. J. Electrochem. Soc. 2017, 164, E3547–E3551. 10.1149/2.0521711jes.

[ref50] Van-Brunt et al. express their flux laws in terms of electroneutral Onsager diffusivities in EMC, designated , , and . Their expressions in terms of Stefan–Maxwell coefficients reveal to leading order that , , and . These scalings were incorporated into the coefficients defined here: , , and . Also Van-Brunt et al. express their flux laws in terms of migration coefficients for EC and salt, ξ_o_ and ξ_e_, respectively. As well as establishing that ξ_o_ ∝ *c*_o_ to leading order, they show that and .

[ref51] Generally 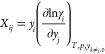 where γ_*i*_ is the activity coefficient of component *i* on a particle-fraction basis and the thermodynamic derivative is evaluated in such a way that *y*_0_ is varied to compensate changes in *y*_o_ or *y*_e_. In ideal solutions, all the γ_*i*_ values are constant.

[ref52] Note that Wang et al. use a thermodynamic factor they call χ, which includes both the ideal and nonideal contributions to mixing energy and is scaled by the total salt stoichiometry. This relates to *X*_ee_ through 2χ = 2 + *X*_ee_.

[ref53] Density measurements reported by Wang et al.,^31^ which are provided in the Supporting Information, were interpolated and combined with Wang’s reported concentration profiles to calculate the particle fractions of EC and LiPF_6_ in the bulk and at the electrode surfaces in their Hittorf cell. The resulting values were then fed to the Δ*U* function in eq 17 to quantify overpotentials.

[ref54] To model the expected Nernstian dependence of open-circuit potential on salt concentration more clearly, one can use 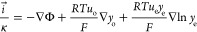 in place of MacInnes eq 9, and treat the lumped quantity *u*_e_*y*_e_ as a composition-dependent material parameter. Inspection of eq 10 suggests that the coefficient of ∇*y*_o_ is not smoothed by a similar transformation.

[ref55] Wang et al. defined the migration flux of EC to be negative in the direction of the conventional current, whereas the opposite convention was used by Van-Brunt et al.,^35^ hence the sign switch between their Ξ and our Ξ̃.

