# Supplementary material for: Overpotential from Cosolvent Imbalance in Battery Electrolytes: LiPF6 in EMC:EC
Source: ACS Omega. 2023 May 26;8(23):21133–44. doi: 10.1021/acsomega.3c02088 (PMC10268269; doi:10.1021/acsomega.3c02088)
Supplement: Supplementary file 1 — ao3c02088_si_001.pdf [file ao3c02088_si_001.pdf]

Supporting Information

Overpotential from cosolvent imbalance in battery electrolytes: $LiPF_6$ in EMC:EC

Taeho Jung[a,b], Andrew A. Wang[c], and Charles W. Monroe[a,b,*]

[a] *Department of Engineering Science, University of Oxford, Parks Road, Oxford, OX1 3PJ, United Kingdom*

[b] *The Faraday Institution, Harwell Campus, Didcot, OX11 0RA, United Kingdom*

[c] *Department of Chemical Engineering, Columbia University, New York, NY 10027, United States of America*

* E-mail: charles.monroe@eng.ox.ac.uk

S1. Raw liquid-junction potential measurement data

Table S1a. EMC:EC = 7:3 mass ratio with $y_o^{ref} = 0.249805$ and $y_e^{ref} = 0.128585$.

y_o^{test}	y_e^{test}	ΔU (mV)			
		Run 1	Run 2	Run 3	Run 4
0.334936	0.00201116	-193.9	-195.51	-194.55	-195.0
0.331892	0.00653695	-147.05	-145.91	-146.16	-146.5
0.318331	0.0266993	-106.39	-107.04	-105.13	-106.3
0.297815	0.0572036	-74.95	-72.04	-70.34	-72.14
0.284403	0.0771436	-53.27	-52.31	-54.73	-54.47
0.269155	0.0998158	-32.18	-32.34	-31.88	-32.98
0.252723	0.124247	-6.93	-8.93	-9.06	-8.035
0.249805	0.128585	0			
0.234636	0.151139	22.08	23.51	24.26	23.16

Table S1b. EMC:EC = 1:1 mass ratio with $y_o^{ref} = 0.456376$ and $y_e^{ref} = 0.0787517$.

y_o^{test}	y_e^{test}	ΔU (mV)			
		Run 1	Run 2	Run 3	Run 4
0.532733	0.00827132	-107.2	-108.0	-106.9	-108.8
0.496650	0.0415770	-45.8	-44.8	-44.3	-44.7
0.456376	0.0787517	0			
0.418035	0.114142	35.85	36.12	35.3	36.0
0.381605	0.147768	85.17	85.33	85.27	85.63

Table S1c. EMC:EC = 3:7 mass ratio with $y_o^{ref} = 0.621185$ and $y_e^{ref} = 0.0772349$.

y_o^{test}	y_e^{test}	ΔU (mV)			
		Run 1	Run 2	Run 3	Run 4
0.723204	0.00780305	-105.0	-109.5	-107.1	-108.0
0.677397	0.0389784	-42.39	-43.32	-42.88	-41.74
0.621185	0.0772349	0			
0.570355	0.111829	35.17	32.1	32.66	34.05
0.522070	0.144690	77.94	78.43	78.25	78.85

Table S1d. Liquid-junction potentials along A_1-B_1-C_1-D_1 and A_2-B_2-C_2-D_2. See Table 2 for the probed solution compositions.

Ref	Test	ΔU (mV)					
		Run 1	Run 2	Run 3	Run 4	Run 5	Run 6
A_1	B_1	8.52	8.10	8.86	8.00	8.945	7.90
A_1	C_1	22.25	22.55	22.20	22.45	22.25	22.15
A_1	D_1	55.20	54.35	54.54	59.02	59.36	59.33
B_1	C_1	14.02	13.93	13.86	13.43	13.33	13.26
B_1	D_1	50.20	50.20	50.21	48.93	49.20	49.17
C_1	D_1	35.22	35.42	35.15	35.31	35.22	35.22
A_2	B_2	15.16	14.98	14.94	14.79	14.87	14.9
A_2	C_2	39.62	39.3	39.02	40.66	40.64	40.64
A_2	D_2	133.45	133.6	132.9	131.8	131.85	131.84
B_2	C_2	23.93	23.85	23.78	23.93	23.88	23.81
B_2	D_2	115.35	115.84	115.94	116.84	116.9	117.07
C_2	D_2	90.75	90.05	89.7	91.7	92.06	92.73

S2. Liquid-junction potentials relative to a common reference composition: $y_\mathrm{o}^\mathrm{ref} = 0$ and $y_\mathrm{e}^\mathrm{ref} = 0.09379$.

Table S2a. EMC:EC = 7:3 mass ratio

$y_\mathrm{o}^\mathrm{test}$	$y_\mathrm{e}^\mathrm{test}$	ΔU (mV)			
		Run 1	Run 2	Run 3	Run 4
0.249805	0.128585	6.33			
0.334936	0.002011	-187.57	-189.18	-188.22	-188.67
0.331892	0.006537	-140.72	-139.58	-139.83	-140.17
0.318331	0.026699	-100.06	-100.71	-98.80	-99.97
0.297815	0.057204	-68.62	-65.71	-64.01	-65.81
0.284403	0.077144	-46.94	-45.98	-48.40	-48.14
0.269155	0.099816	-25.85	-26.01	-25.55	-26.65
0.252723	0.124247	-0.60	-2.60	-2.73	-1.71
0.234636	0.151139	28.41	29.84	30.59	29.49

Table S2b. EMC:EC = 1:1 mass ratio

$y_\mathrm{o}^\mathrm{test}$	$y_\mathrm{e}^\mathrm{test}$	ΔU (mV)			
		Run 1	Run 2	Run 3	Run 4
0.456376	0.078752	-58.63			
0.532733	0.008271	-165.83	-166.63	-165.53	-167.43
0.496650	0.041577	-104.43	-103.43	-102.93	-103.33
0.418035	0.114142	-22.78	-22.51	-23.33	-22.63
0.381605	0.147768	26.54	26.70	26.64	27.00

Table S2c. EMC:EC = 3:7 mass ratio

$y_\mathrm{o}^\mathrm{test}$	$y_\mathrm{e}^\mathrm{test}$	ΔU (mV)			
		Run 1	Run 2	Run 3	Run 4
0.621185	0.077235	-68.22			
0.723204	0.007803	-173.22	-177.72	-175.32	-176.22
0.677397	0.038978	-110.61	-111.54	-111.10	-109.96
0.570355	0.111829	-33.05	-36.12	-35.56	-34.17
0.522070	0.144690	9.72	10.21	10.03	10.63

Table S2d. Across A_1-ref, B_1-ref, and C_1-ref.

$y_\mathrm{o}^\mathrm{test}$	$y_\mathrm{e}^\mathrm{test}$	ΔU (mV)					
		Run 1	Run 2	Run 3	Run 4	Run 5	Run 6
0.598516	0.0901736	-56.56	-55.71	-55.90	-60.38	-60.72	-60.69
0.443758	0.0899525	-51.56	-51.56	-51.57	-50.29	-50.56	-50.53
0.275268	0.0897390	-36.58	-36.78	-36.51	-36.67	-36.58	-36.58

Table S2e. Across A_2-ref, B_2-ref, and C_2-ref.

$y_\mathrm{o}^\mathrm{test}$	$y_\mathrm{e}^\mathrm{test}$	ΔU (mV)					
		Run 1	Run 2	Run 3	Run 4	Run 5	Run 6
0.719059	0.0101935	-161.10	-161.25	-160.55	-159.45	-159.50	-159.49
0.526531	0.0102671	-143.00	-143.49	-143.59	-144.49	-144.55	-144.72
0.327312	0.0100977	-118.40	-117.70	-117.35	-119.35	-119.71	-120.38

S3. Density vs. composition at 25.00 ± 0.02 °C

Mass densities ρ of EMC:EC:$LiPF_6$ solutions were reported as functions of the EC and electrolyte mass fractions, ω_o and ω_e, respectively, by Wang et al. [1]. Mass fractions can be converted directly to particle fractions y_o and y_e with the formulas

$$y_\mathrm{o} = \frac{\frac{\bar{m}_0}{\bar{m}_\mathrm{o}}\omega_\mathrm{o}}{1+\left(\frac{\bar{m}_0}{\bar{m}_\mathrm{o}}-1\right)\omega_\mathrm{o}+\left(\frac{2\bar{m}_0}{\bar{m}_\mathrm{e}}-1\right)\omega_\mathrm{e}} \quad \text{and} \quad y_\mathrm{e} = \frac{\frac{\bar{m}_0}{\bar{m}_\mathrm{e}}\omega_\mathrm{e}}{1+\left(\frac{\bar{m}_0}{\bar{m}_\mathrm{o}}-1\right)\omega_\mathrm{o}+\left(\frac{2\bar{m}_0}{\bar{m}_\mathrm{e}}-1\right)\omega_\mathrm{e}},$$

in which $\bar{m}_0$, $\bar{m}_\mathrm{o}$, and $\bar{m}_\mathrm{e}$ are the molar masses of the EMC, EC, and $LiPF_6$ components, respectively. The total species concentration c_T is then given by

$$c_\mathrm{T} = \frac{\rho}{\bar{m}_\mathrm{o}y_\mathrm{o}+\bar{m}_\mathrm{e}y_\mathrm{e}+\bar{m}_0(1-y_\mathrm{o}-2y_\mathrm{e})},$$

and the component molarities by $c_\mathrm{o} = c_\mathrm{T}y_\mathrm{o}$ and $c_\mathrm{e} = c_\mathrm{T}y_\mathrm{e}$.

Table S3. Density measurement at 25°C at EMC:EC mass ratio of 1:0, 7:3, 1:1, and 3:7.

ω_o	ω_e	ρ (g/cm^3)	y_o	y_e	c_o (M)	c_e (M)	c_T (M)
0	0	1.0061	0	0	0	0	9.66428
0	0.01394	1.0177	0	0.00950	0	0.09339	9.82622
0	0.07384	1.0674	0	0.04926	0	0.51886	10.5337
0	0.13444	1.1176	0	0.08776	0	0.98911	11.2703
0	0.19654	1.1691	0	0.12555	0	1.51262	12.0481
0	0.24616	1.2102	0	0.15460	0	1.96111	12.6855
0	0.04389	1.0425	0	0.02960	0	0.30121	10.1768
0	0.10414	1.0925	0	0.06872	0	0.74897	10.8993
0	0.16549	1.1433	0	0.10686	0	1.24555	11.6558
0	0.22135	1.1897	0	0.14020	0	1.73358	12.3655
0.3	0	1.0948	0.33627	0	3.72965	0	11.0911
0.29699	0.01003	1.1024	0.33190	0.00650	3.71786	0.07279	11.2016
0.28117	0.06277	1.1428	0.30935	0.04004	3.64881	0.47223	11.7951
0.26326	0.12247	1.1885	0.28465	0.07677	3.55300	0.95820	12.4821
0.23944	0.20188	1.2493	0.25308	0.12370	3.39684	1.66031	13.4218
0.22532	0.24892	1.2854	0.23504	0.15053	3.28889	2.10633	13.9932
0.28908	0.0364	1.1226	0.32054	0.02340	3.68514	0.26900	11.4967
0.27221	0.09262	1.1657	0.29689	0.05856	3.60332	0.71075	12.1370
0.25135	0.16218	1.2189	0.26869	0.10050	3.47903	1.30135	12.9483
0.23238	0.2254	1.2674	0.24400	0.13720	3.34444	1.88060	13.7068
0.5	0	1.1573	0.54174	0	6.57094	0	12.1293
0.49307	0.01387	1.1679	0.53234	0.00868	6.53922	0.10664	12.2839
0.46655	0.0669	1.2084	0.49698	0.04131	6.40207	0.53219	12.8819
0.43562	0.12876	1.2556	0.45691	0.07829	6.21113	1.06429	13.5937
0.40524	0.18952	1.3021	0.41874	0.11353	5.99195	1.62453	14.3096
0.37548	0.24904	1.3475	0.38242	0.14704	5.74549	2.20915	15.0239
0.47981	0.04039	1.1881	0.51454	0.02511	6.47342	0.31590	12.5810
0.45109	0.09783	1.232	0.47680	0.05995	6.31081	0.79343	13.2359
0.42043	0.15914	1.2789	0.43768	0.09604	6.10579	1.33981	13.9503
0.39036	0.21928	1.3248	0.40045	0.13041	5.87255	1.91239	14.6649
0.7	0	1.2263	0.73393	0	9.74779	0	13.2816
0.69055	0.0135	1.2364	0.72192	0.00818	9.69540	0.10988	13.4300
0.65472	0.06468	1.2746	0.67701	0.03877	9.47635	0.54272	13.9973
0.609	0.13	1.3234	0.62111	0.07686	9.15208	1.13256	14.7351
0.56651	0.1907	1.3688	0.57051	0.11133	8.80560	1.71838	15.4346
0.5251	0.24986	1.413	0.52241	0.14411	8.42550	2.32416	16.1283
0.67264	0.03909	1.2555	0.69935	0.02356	9.58983	0.32308	13.7125
0.63186	0.09734	1.299	0.64887	0.05795	9.32055	0.83239	14.3643
0.58776	0.16035	1.3461	0.59565	0.09421	8.98439	1.42094	15.0833
0.5458	0.22028	1.3909	0.54631	0.12782	8.62067	2.01697	15.7799

References

[1] A.A. Wang, S. Greenbank, G. Li, D.A. Howey, and C.W. Monroe, "Current-driven solvent segregation in lithium-ion electrolytes." *Cell Reports Physical Science* **3**:9 (2022) 101047.
